# Positive predictive values of fecal immunochemical tests used in the STOP CRC pragmatic trial

**DOI:** 10.1002/cam4.1727

**Published:** 2018-08-13

**Authors:** Carrie M. Nielson, Amanda F. Petrik, Lorie Jacob, William M. Vollmer, Erin M. Keast, Jennifer L. Schneider, Jennifer S. Rivelli, Tanya J. Kapka, Richard T. Meenan, Rajasekhara R. Mummadi, Beverly B. Green, Gloria D. Coronado

**Affiliations:** ^1^ Kaiser Permanente Center for Health Research Portland Oregon; ^2^ OCHIN Portland Oregon; ^3^ Kaiser Permanente Washington Health Research Institute Seattle Washington

**Keywords:** cancer, colorectal, fecal immunochemical test, neoplasia, screening

## Abstract

Annual fecal immunochemical testing (FIT) is cost‐effective for colorectal cancer (CRC) screening. However, FIT positivity rates and positive predictive value (PPV) can vary substantially, with false‐positive (FP) results adding to colonoscopy burden without improving cancer detection. Our objective was to describe FIT PPV and the factors associated with FP results among patients undergoing CRC screening. In an ongoing pragmatic clinical trial of mailed‐FIT outreach, clinics delivered one of three FIT brands (InSure, OC‐Micro, and Hemosure). Patients who had a positive FIT result and a follow‐up colonoscopy were included in this analysis (N = 1130). Patients’ demographic and medical histories were abstracted from electronic health records (EHR). Associations with a FP result (ie, a positive FIT result with no evidence of advanced neoplasia during follow‐up colonoscopy) were evaluated for FIT brand and patient factors using mixed‐effects multivariable logistic regression. The mean proportion of FIT‐positive results ranged from 8% in centers using the OC‐Micro test to 21% for Hemosure. PPVs for advanced neoplasia were 0.30 to 0.17, respectively (*P* for *χ* ^2^ = 0.08). In multivariable‐adjusted models, use of Hemosure was associated with greater odds of a FP result than OC‐Micro (OR = 2.00, 95% CI: 0.47‐8.56) or InSure (OR = 1.72, 95% CI: 0.44‐6.68). However, only female sex (OR = 1.58, 95% CI: 1.19‐2.10) and history of a colorectal condition (OR = 2.17, 95% CI: 1.13‐4.15) were significantly associated with FP. In conclusion, FIT positivity varied by brand, and FP results differed by patient factors available through the EHR. These results can be used to minimize the frequency of FP results, reducing patient distress and colonoscopy burden.

## 1 INTRODUCTION

While colorectal cancer (CRC) screening is strongly recommended for adults ages 50 to 75 years,^1^, ^2^ compliance with screening among all but the highest family income level (≥600% of federal poverty level) remains below the Healthy People 2020 target of 70%.^3^, ^4^ Compliance in Federally Qualified Health Centers (FQHCs) is especially low, estimated at 39.9%.^5^ Indeed, fewer than 30% of uninsured patients are up to date on screening.^6^ Annual fecal testing, through methods including the fecal immunochemical test (FIT), is a relatively accessible screening method with minimal adverse outcomes,^7^ but optimal test performance is required to ensure that CRC is prevented or detected at an early stage. Optimal test performance also minimizes unnecessary follow‐up among false positives (FPs). Minimizing FP FIT results could reduce preventable patient worry and stress,^8^, ^9^, ^10^, ^11^ as well as adverse events and costs that can occur with follow‐up colonoscopy.

Despite advances in the accuracy of fecal tests,^12^, ^13^, ^14^ FP results outnumber true‐positive (TP) results. Factors affecting FP include choice of FIT, number of samples collected per screening round, and—for quantitative FITs—hemoglobin concentration threshold for determining a positive test.^15^, ^16^ Varying the concentration of hemoglobin used as a threshold for positivity at values between 25 ng/mL and 200 ng/mL using an OC‐Micro system can result in FIT positivity rates that range from 18% to 5%,^17^, ^18^ which has substantial influence on follow‐up colonoscopy demand.^19^ Even at the same threshold for positivity, test brand may affect sensitivity and positive predictive value (PPV).^20^

Patient characteristics, such as age, sex, smoking status, medication use, and comorbidities,^15^, ^19^, ^21^, ^22^, ^23^, ^24^, ^25^, ^26^ have been shown to affect fecal hemoglobin concentration and test performance. Because PPV improves as prevalence of disease rises, the FIT PPV for CRC can range from 1.5% in the 50‐54 age range to 9% in the 70‐74 range for the same hemoglobin threshold, and it has been suggested that using higher thresholds at younger ages significantly increases diagnostic yield.^27^ Similar arguments have been made for sex‐specific cutoffs because of the higher prevalence of CRC in men than women.^26^, ^28^, ^29^

In this study, we describe FIT positivity rates and follow‐up colonoscopy results among FQHCs participating in the Strategies and Opportunities to Stop Colon Cancer in Priority Populations (STOP CRC) study. PPVs and factors associated with a false‐positive FIT result were evaluated to elucidate potential fecal test strategies that might minimize FP results and the associated patient and provider burden.

## 2 METHODS

STOP CRC is a multicenter pragmatic study of colon cancer screening in FQHC clinics in Oregon and Washington state. STOP CRC was designed to test the use of a direct mail approach to CRC screening as compared to usual care.^30^ The Institutional Review Board of Kaiser Permanente Northwest (KPNW) approved all study activities, and participating clinics ceded human subjects review authority to this IRB. The trial is registered at ClinicalTrials.gov (NCT01742065). The current analysis of FIT performance was undertaken after observations of wide ranges in FIT positivity across health centers and was not part of the original trial design.

### 2.1 Patient eligibility

Patients were eligible for STOP CRC if they (a) were 50‐74 years old, (b) had visited their clinic in the previous year, and (c) were due for CRC screening. Patients were due for screening if there was no evidence in the electronic health record (EHR) of (a) a fecal test in the previous year, (b) an order for a fecal test in the previous 6 months, (c) a flexible sigmoidoscopy in the previous 4 years, (d) a colonoscopy in the previous 9 years, or (e) an order for a sigmoidoscopy/colonoscopy in the previous year. Patients were excluded if there was EHR evidence of any of several health conditions that made them poor candidates for fecal testing (eg, history of CRC, inflammatory bowel disease, or end‐stage renal failure).^31^

### 2.2 Analytic sample

**Figure 1:**
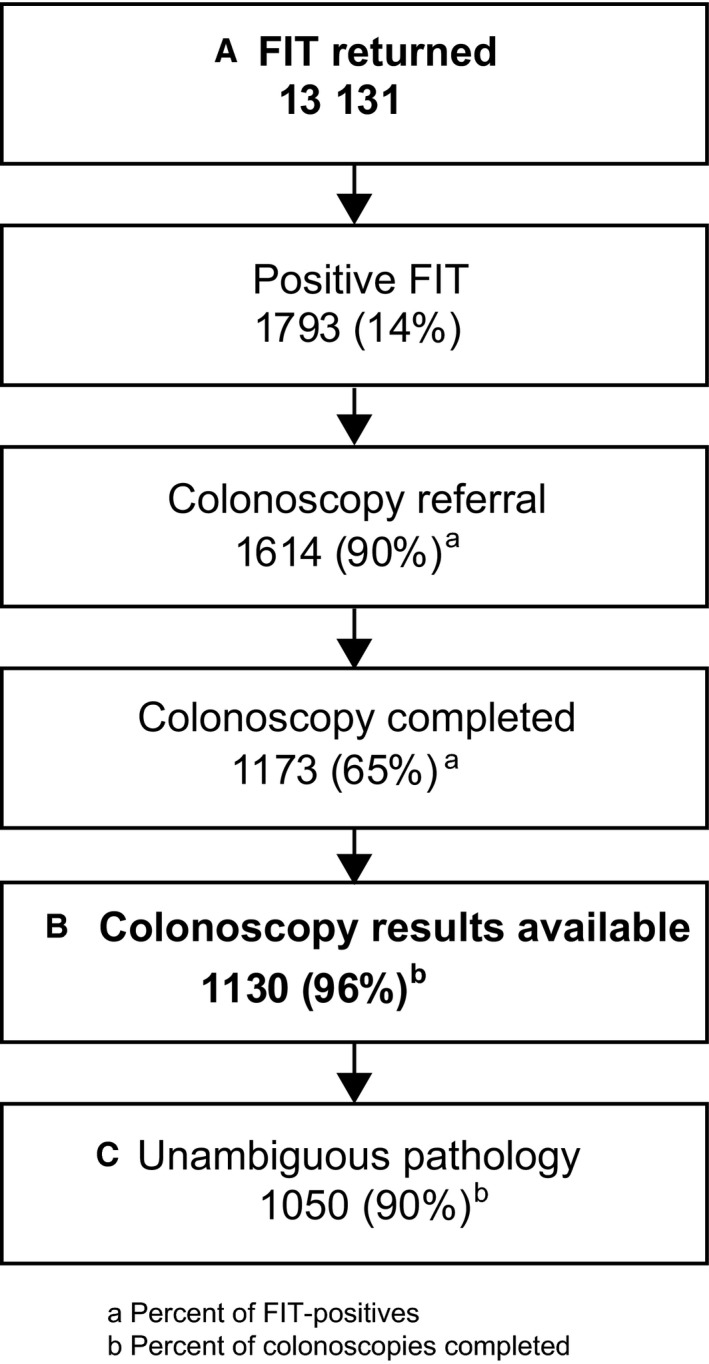
Analytic sample for report of FIT positivity rate (A); descriptive results of colonoscopy, by FIT kit (B); and PPV and factors associated with a FP FIT (C)

Patients were included in the analyses of PPV and FP predictors if they had a positive FIT result using an OC‐Micro, InSure, or Hemosure test (details below), completed a follow‐up colonoscopy within 12 months of their result, and had a subsequent colonoscopy procedure report, pathology report, or colonoscopy provider notes with sufficient detail to determine the result (Figure 1).

### 2.3 Fecal test

All but two of the participating clinics used one of three FIT kit brands. (a) The OC‐Micro single‐sample, automated test (Polymedco, Inc., Cortlandt Manor, NY), was processed by one laboratory for four health centers, using a threshold for positivity of 20 μg hHb/g feces. (b) The InSure double‐sample qualitative visual test (Enterix, Inc., Edison, NJ), which has a lower limit of detection of 50 μg hHb/g, was processed by laboratory technicians at a single laboratory for two health centers and in‐house for one health center. (c) A Hemosure single‐sample test, also a qualitative visual test (Hemosure, Inc., Irwindale, CA), was used in one health center; the threshold for positivity was 50 μg hHb/g. Two additional clinics used the Consult Diagnostics iFOBT (PSS World Medical, Inc., Jacksonville, FL); however, the small number of patients and follow‐up colonoscopies (n = 7) precluded its inclusion in this analysis. All tests were mailed from or distributed in the clinics from February 2014 to February 2016, and patients returned the completed tests to their clinic or mailed them to a processing laboratory.

### 2.4 Chart abstraction

Colonoscopy results were determined through chart abstraction of the pathology or procedure report, when available, or through clinician notes. All charts were accessed in collaboration with OCHIN, a nonprofit health center network with an organization‐wide EHR that allows researchers to access clinical and utilization data across all OCHIN clinic sites. A trained abstractor collected data for the fields listed in Table S1.

#### 2.4.1 Case definitions

A result was considered positive for cancer if the pathology report indicated invasive carcinoma. The result was positive for advanced adenoma (AA) if any of the following were found: a traditional serrated, villous, or tubulovillous adenoma of any size; an adenoma of any size with high‐grade dysplasia; a sessile serrated or tubular adenoma >10 mm; or ≥3 serrated or tubular adenomas <10 mm. Nonadvanced adenomas (tubular or serrated adenomas <10 mm) and other polyps were also recorded as their own categories. When a pathology report was unavailable and the provider notes indicated the presence of a polyp but not its size, pathology, or number, we reported the presence of a “polyp of unknown pathology” and excluded the case from primary analyses of PPV.

#### 2.4.2 Chronic conditions

Chronic conditions were ascertained by searching for ICD‐9 or ICD‐10 codes up to 2 years before the FIT result. The prevalence of hypertension and diabetes was described using the ICD‐9 codes for these conditions in Elixhauser's original algorithm.^32^ Morbidities directly related to bleeding (eg, anal fissures and hemorrhoids) and indirectly, through medications known to increase the risk of gastrointestinal bleeding (eg, anticoagulants and NSAIDs), were included. NSAID use was not recorded in the EHR; therefore, we used Evidex (Advera Health Analytics, Santa Rosa, CA) to search for medications showing a high risk of gastrointestinal hemorrhage and their indications. Data cited from Evidex were aggregated, standardized, and curated from the FDA Adverse Events Reporting System (FAERS), spanning November 1997 through December 2016.^33^ All codes used in this analysis are listed in Table S2.

### 2.5 Statistical methods

FIT results reported as positive, negative, or inconclusive/unknown were calculated. Among patients with a positive FIT result, the proportions with a subsequent colonoscopy referral and who completed a colonoscopy were also calculated. Finally, patients with a FIT‐positive result and results from a follow‐up colonoscopy were included in subsequent analyses (Figure 1).

In the main analysis of PPV and false positivity, a “positive” result included findings of cancer or advanced adenoma, and a “negative” result included nonadvanced adenoma, nonadenomatous polyps, and no abnormal findings. In a sensitivity analysis, we considered all adenomas as “positive.” Polyps of unknown pathology were excluded from calculations. In separate sensitivity analyses, we considered these polyps to be, alternately, all “positive” (ie, advanced neoplasia) or all “negative” (ie, normal) colonoscopy findings. To determine the factors associated with a false‐positive colonoscopy outcome (ie, no finding of cancer or advanced adenoma), we used a mixed‐effects logistic regression model (SAS PROC GLIMMIX) with a random effect for health center to account for intracenter correlations in patient characteristics. The adjusted model additionally included FIT kit type and all variables with unadjusted *P* < 0.20.

## 3 RESULTS

Patients who returned a FIT (N = 13 131) were primarily ages 50‐64 (81%) and white (84%); 17% were Hispanic, and 14%‐15% were non‐English speakers (Table 1). Approximately one‐quarter had a diagnosis of type 2 diabetes, and half had been diagnosed with hypertension. Diagnosis of diverticulum, hemorrhoids, or anal fissures was uncommon (2%‐3% for each diagnosis, Table 1), although 5% of those who returned a FIT and 7% of those who completed a colonoscopy had a history of one of the three comorbidities. Patient characteristics were similar for the subset who also had a follow‐up colonoscopy result available, although the proportion of female and Hispanic patients was lower, and the prevalence of smoking and each comorbidity was slightly higher (Table 1).

**Table 1:** Characteristics of patients in the STOP CRC evaluation of FIT positivity and colonoscopy outcomes

	FIT returned (N = 13 131)	Colonoscopy results available (N = 1130)
Age		
50‐64	10 670 (81%)	939 (83%)
65‐74	2461 (19%)	191 (17%)
Female	7435 (57%)	578 (51%)
Hispanic	2244 (17%)	124 (11%)
Non‐white	2032 (16%)	185 (16%)
Language		
English	9410 (72%)	894 (79%)
Spanish	1942 (15%)	92 (8%)
Other	1779 (14%)	144 (13%)
Insurance status		
Medicaid	5344 (41%)	477 (42%)
Medicare	2149 (16%)	197 (17%)
Uninsured	3437 (26%)	300 (27%)
Commercial	1767 (14%)	131 (12%)
Other/Unknown	434 (3%)	25 (2%)
Federal poverty level		
<100%	5353 (41%)	479 (42%)
100%‐150%	2216 (17%)	192 (17%)
>150%	2686 (21%)	209 (19%)
Unknown	2876 (22%)	250 (22%)
Comorbidities		
Diabetes	3184 (24%)	313 (28%)
Hypertension	6584 (50%)	653 (58%)
Diverticulum	244 (2%)	29 (3%)
Hemorrhoids or anal fissures	435 (3%)	50 (4%)
Anticoagulant use	906 (7%)	130 (12%)
NSAIDs use	2851 (22%)	286 (25%)
Tobacco use		
Never	5966 (45%)	411 (36%)
Former	3064 (23%)	298 (26%)
Current	2830 (22%)	301 (27%)
Unknown	1271 (10%)	120 (11%)
Season of FIT return		
Winter	3318 (25%)	325 (29%)
Spring	4090 (31%)	367 (33%)
Summer	2875 (22%)	201 (18%)
Fall	2848 (22%)	237 (21%)

**Figure 2:**
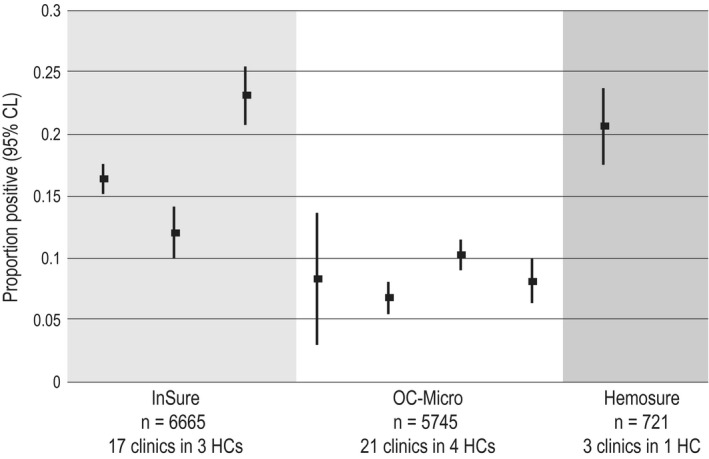
Mean (95% CI) proportion of FITs that were positive, by health center and kit type (N = 13 131)

FIT positivity was 21% in the health center that used Hemosure kits, 12%‐23% in centers using InSure, and 7%‐10% in centers that used OC‐Micro (Figure 2). Many more inconclusive results were found in the center that used Hemosure (9%) than in those using InSure or OC‐Micro (all centers ≤1%). Among patients with a positive FIT result and completed colonoscopy, 14% who used Hemosure had advanced neoplasia, defined as CRC or AA (PPV = 0.17), compared with 26% who used InSure and 29% who used OC‐Micro (PPV = 0.27 and 0.30, respectively, *P* for *χ* ^2^ across three kit types = 0.08, Table 2). In the sensitivity analysis, PPVs for CRC or any adenoma were 0.39 for Hemosure, 0.53 for InSure, and 0.44 for OC‐Micro (*P* for *χ* ^2^ across three kit types = 0.003, Figure S1). When polyps of unknown pathology were included and considered positive for advanced neoplasia, PPVs were uniformly higher, but the ranking across kits remained the same; when these polyps were included in PPV calculations and considered as normal colonoscopy findings, results were nearly identical to our primary analysis (results not shown).

**Table 2:** Most advanced colonoscopy result by FIT kit type, among those with a positive FIT result and completed colonoscopy (N = 1130)^a^

	InSure (n = 718)	OC‐Micro (n = 329)	Hemosure (n = 83)
Advanced neoplasia	186 (26%)	96 (29%)	12 (14%)
Colorectal cancer	12 (2%)	11 (3%)	1 (1%)
Advanced adenoma	174 (24%)	85 (26%)	11 (13%)
No advanced neoplasia	491 (68%)	225 (68%)	60 (72%)
Nonadvanced adenoma	176 (25%)	45 (14%)	16 (19%)
Nonadenomatous polyp	65 (9%)	32 (10%)	10 (12%)
No polyp or adenoma	246 (34%)	132 (40%)	34 (41%)
Polyp of unknown pathology^b^	45 (6%)	24 (7%)	11 (13%)
PPV for advanced neoplasia (95% CI)^c^	0.27 (0.24‐0.31)	0.30 (0.25‐0.35)	0.17 (0.08‐0.25)

a Sample comprises patients with an abnormal FIT result who were referred for a follow‐up colonoscopy and who had evidence of colonoscopy completion in their electronic medical record.

b Pathology report was unavailable in the patient's health record; therefore, the presence of polyp(s) was determined through provider notes. This category was not included in PPV calculations.

c PPV was calculated as advanced neoplasia/(advanced neoplasia + no advanced neoplasia). *P* for *χ* ^2^ = 0.08.

Factors significantly associated with a false‐positive result in bivariate analyses were female sex, insurance status, and history of any colorectal morbidity (Table 3). In addition, language and NSAID use were associated with a false positive with *P* < 0.20 and were carried forward to the multivariable model. In the full model, female sex and colorectal morbidity were significantly associated with a FP test, independently of other measured variables, including FIT kit brand, language, insurance status, and NSAID use. Results were similar for the sensitivity analysis in which nonadvanced adenomas were considered a “positive” colonoscopic finding (Table S3).

**Table 3:** Factors associated with a false‐positive FIT (ie, no evidence of advanced neoplasia through colonoscopy after a positive FIT), N = 1027

	Unadjusted^a^			Adjusted^a^		
	OR	95% CI	*P*	OR	95% CI	*P*
FIT type						
Hemosure vs InSure	1.86	0.50‐6.15	0.32	1.72	0.44‐6.68	0.47
OC‐Micro vs InSure	0.84	0.41‐1.71		0.85	0.37‐1.97	
Hemosure vs OC‐Micro	2.23	0.67‐7.46		2.00	0.47‐8.56	
Age						
50‐64	Ref		0.73			
65‐74	1.07	0.74‐1.54				
Female	1.71	1.30‐2.26	0.0002	1.59	1.20‐2.11	0.001
Hispanic	1.13	0.72‐1.79	0.59			
Non‐white	1.10	0.75‐1.61	0.63			
Language						
English	Ref		0.17	Ref		0.47
Spanish	1.71	0.97‐3.03		1.44	0.80‐2.60	
Other	1.15	0.74‐1.78		1.09	0.69‐1.70	
Insurance status						
Medicaid	Ref		0.03	Ref		0.09
Medicare	0.84	0.58‐1.22		0.82	0.56‐1.20	
Uninsured	1.51	1.07‐2.15		1.40	0.98‐2.01	
Commercial	1.10	0.68‐1.78		1.08	0.66‐1.76	
Federal poverty level						
<100%	Ref		0.87			
100%‐150%	0.89	0.60‐1.32				
>150%	0.90	0.62‐1.32				
Unknown	1.04	0.72‐1.49				
Comorbidities						
Diabetes	1.18	0.86‐1.60	0.30			
Hypertension	1.09	0.82‐1.43	0.56			
Colorectal condition^b^	2.19	1.16‐4.14	0.02	2.18	1.14‐4.18	0.02
Anticoagulant use	0.98	0.64‐1.50	0.92			
NSAIDs use	1.26	0.92‐1.75	0.16	1.17	0.83‐1.62	0.37
Tobacco use						
Never	Ref		0.37			
Former	0.88	0.62‐1.07				
Current	0.76	0.54‐1.07				
Unknown	1.08	0.65‐1.79				
Season of FIT return						
Winter	Ref		0.34			
Spring	0.83	0.59‐1.18				
Summer	1.10	0.73‐1.68				
Fall	1.14	0.76‐1.71				

CI, confidence interval; OR, odds ratio.

a All models include a random effect for health center, nested within FIT brand, to account for any unmeasured population differences across center. The adjusted model additionally includes FIT kit type and all variables with unadjusted *P* < 0.20.

b From diagnosis codes present in the EHR within 2 years prior to FIT eligibility (Table S2).

## 4 DISCUSSION

We observed a broad range of FIT positivity rates by health center. This has implications for evaluating and planning screening strategies, including resources for follow‐up colonoscopy for FIT‐positive patients. Although models of cost‐effectiveness use FIT positivity parameters ≤10%,^34^, ^35^ four (50%) clinics had positivity rates over 10%. Higher rates can raise the overall cost of screening, but their impact on cost‐effectiveness remains to be evaluated. The positivity rate observed for some centers was much higher than predicted by our previous experience with OC‐Micro, a kit with extensive test performance literature. However, qualitative FITs such as InSure and Hemosure have a wide range of average positivity rates (6%‐47%)^36^ and little published research to elucidate the factors affecting false positivity. Positivity rates have been observed to decline in subsequent years after FIT screening is introduced into a population.^37^ Therefore, considering the baseline screening participation rates in a clinic system might also inform expected FIT positivity rates.

PPV for advanced neoplasia (including CRC or AA) also varied substantially (16% for the clinic that used Hemosure to 31% for those using OC‐Micro). These values are similar to those previously reported for advanced neoplasia^26^ and are between those reported for CRC (2%‐17%)^15^, ^26^ and advanced adenoma (35%‐51%).^37^, ^38^ Moreover, in a sensitivity analysis that moved nonadvanced adenomas into the “positive” outcome, PPVs rose to 39‐53%. The substantial number of nonadvanced adenomas detected in clinics that used the InSure FIT led to a PPV that was significantly higher than with Hemosure or OC‐Micro. However, in multivariable analyses of false positivity, differences by FIT kit were not statistically significant. The large differences in PPV across definitions of a “positive” colonoscopic finding, both in our study and in previous literature, underscore the need to interpret screening test value in the context of follow‐up diagnostic and treatment measures appropriate to each specific outcome (CRC, AA, and nonadvanced adenoma).

Several patient factors have been associated with FIT positivity and false positivity. In our analysis, women with a FIT‐positive result were about 50% more likely to have no evidence of advanced neoplasia than FIT‐positive men, which is similar to previous reports.^39^, ^40^ Although other studies have reported no sex differences in FP,^21^, ^23^ sex‐specific hemoglobin concentration cutoffs have been suggested. In a large study that evaluated PPV for advanced neoplasia at varying hemoglobin concentration cutoffs, the PPVs achieved for women (33%‐43%) were lower than for men (53%‐63%) at every hemoglobin cutoff.^26^ Only among women over 65 did PPVs exceed 40% at most hemoglobin cutoff values,^26^ supporting the importance of age and sex in optimizing screening strategies.^28^ Reported effects of age on false positivity have been inconsistent,^21^, ^23^, ^39^, ^41^, ^42^ and in this study, we saw no association.

As in previous studies, we observed that a history of colorectal conditions was associated with false positivity. Although each condition was uncommon in the study sample, the prevalence of any was 5% and doubled the odds of a FP FIT. These findings support previous associations with false positivity reported for the presence of hemorrhoids (ORs 1.1‐2.9), diverticula (OR = 1.9), and anal fissure (OR = 3.7).^23^, ^41^, ^42^, ^43^ Although these conditions can be discovered during colonoscopy, we relied on diagnosis codes at the time of FIT eligibility determination to ascertain them, and the symptoms and comorbidities that led to the diagnoses are unknown. Colonoscopy results for findings other than polyps, adenomas, and cancer were not collected in this study. Because we did not consider new findings of gastrointestinal disease discovered during the follow‐up colonoscopy, the true prevalence is expected to be higher.^44^

Although smoking has been consistently associated with false positivity (ORs from 1.3 to 1.7),^21^, ^23^, ^24^ we saw no association. Smoking history was determined from social history fields in the EHR, and a substantial proportion (10%) were missing data on smoking status. The potential for misclassification and the effect of missing values may have affected our ability to detect an association.

Use of NSAIDs was not significantly associated with false positivity, which may be due to a lower CRC risk among NSAID users.^45^ Previous studies have reported significant associations between use of antiplatelet medication (OR for false positivity ≈2.5)^23^ or proton‐pump inhibitors (OR = 1.8).^42^ We found no association between anticoagulant use and false positivity. Previous studies showed no negative impact of Warfarin on FIT test performance,^46^, ^47^ while low‐dose aspirin was suggested to improve sensitivity.^48^ In considering the body of evidence for anticoagulant use on FIT performance, the US Multi‐Society Task Force on Colorectal Cancer found no rationale for altering anticoagulant medication before FIT screening to improve PPV.^2^

Estimating FIT positivity rates allows for prediction of colonoscopy service demands; however, the challenges to health systems extend beyond knowing this rate. Given current uncertainty about FIT accuracy, a health system considering implementation of a FIT screening program must weigh the costs and benefits of strategies that lead to greater or lesser use of colonoscopy. We have previously reported on interviews we conducted with 36 leaders in the health centers included in this analysis.^49^ Among the factors they considered important in deciding which FIT brand to use, the most common was “quality of results/better test performance,” mentioned by one‐third of respondents. Clearly, there is demand for comparative test performance data. Until test accuracy is optimized for population screening, health systems with limited budgets must balance the competing risks of delays in disease recognition and potential overuse of colonoscopy resources. More research is critical for understanding how best to allocate limited screening resources.

This study has notable limitations. Each clinic selected a single FIT kit when the STOP CRC study started, and head‐to‐head comparisons of positivity rates or other test performance characteristics, including false‐negative rates and negative predictive value, were not included in the study design. We acknowledge the possibility of differences in laboratory handling of returned FITs, variability in colonoscopy quality, unmeasured population or provider characteristics, and environmental factors that could explain the differences observed in positivity and PPV. Studies that use colonoscopy as the reference or gold standard have been carried out for the OC FIT‐CHEK family of tests and InSure, demonstrating sensitivity for detecting advanced neoplasia that was superior to the guaiac‐based stool tests.^50^ We are not aware of any such studies that included the Hemosure FIT. The sample available in this study was small; future studies of larger representative samples are needed for confirmation and improved precision of estimates. In vitro spike‐in proficiency tests have demonstrated high sensitivity and specificity (all ≥93%) for hemoglobin in all three tests used in our study^51^; however, such tightly controlled studies may not translate into similarly high‐performance characteristics in real‐world settings of variable sample preparation, handling, and analysis. Our analysis represents real‐world performance of the FITs that clinics chose to use, and results are intended to inform future FIT choices and the comparative cost‐effectiveness of each.

This study has implications for CRC screening research and implementation. The quality of fecal test results is important. However, given the lack of test performance data for the most commonly used test among these providers (InSure), the need for better population‐based test performance information and communication of that information to providers is apparent. Although several well‐controlled comparisons of FIT performance have been reported, the need for performance assessment in real‐world clinical settings has been noted (eg, Ref 16). Forecasting colonoscopy burden depends on reliable estimates of expected positivity rate. Subpopulation‐specific screening recommendations (eg, by sex and comorbidity) could help ensure that patients with a high risk of false‐positive FIT are offered appropriate screening and diagnostic follow‐up care.

## CONFLICT OF INTEREST

None declared.
