# Supplementary figures and images for: Positive predictive values of fecal immunochemical tests used in the STOP CRC pragmatic trial
Source: Cancer Med. 2018 Aug 13;7(9):4781–90. doi: 10.1002/cam4.1727 (PMC6144161; doi:10.1002/cam4.1727)

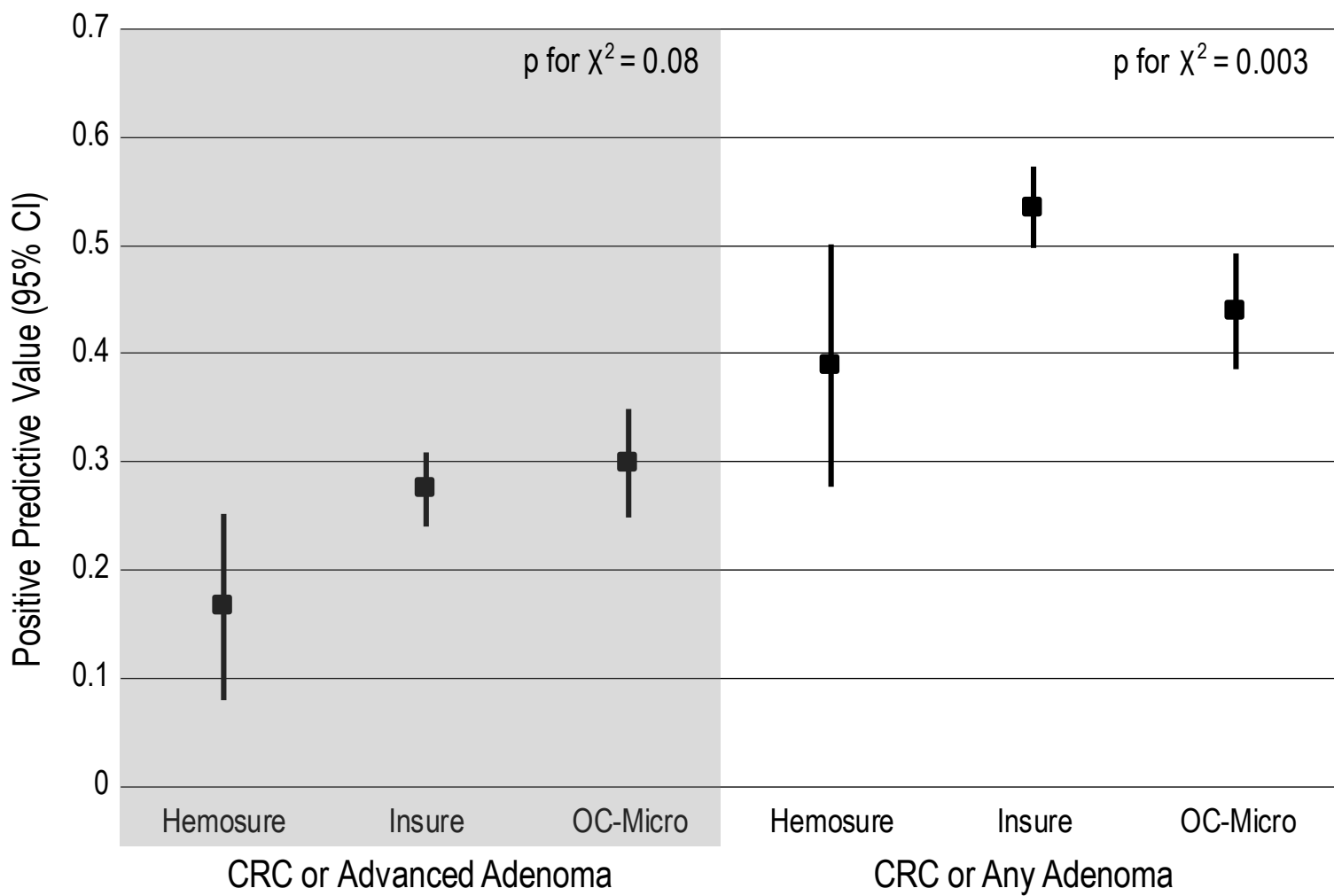

Supplement: Supplementary file 1 [file CAM4-7-4781-s001.pdf]
