# Supplementary material for: Positive predictive values of fecal immunochemical tests used in the STOP CRC pragmatic trial
Source: Cancer Med. 2018 Aug 13;7(9):4781–90. doi: 10.1002/cam4.1727 (PMC6144161; doi:10.1002/cam4.1727)
Supplement: Supplementary file 2 [file CAM4-7-4781-s002.docx]

**SUPPLEMENTAL MATERIALS**

Tables and Figure

[Supplemental Table 1. Chart abstraction fields 23](#_Toc505158694)

[Supplemental Table 2. Diagnosis codes (ICD-9 or -10) used 26](#_Toc505158695)

[Supplemental Table 3. Factors associated with a false-positive FIT (i.e., no evidence of advanced neoplasia or any adenoma), N=1,027 27](#_Toc505158696)

[Supplemental Figure 1. PPVs by FIT type for the main outcome of advanced neoplasia (left) and for the sensitivity analysis that included any adenoma (right) 28](#_Toc505158697)

# Supplemental Table 1. Chart abstraction fields

| **Header Questions** | |
| --- | --- |
| 1a | **Study ID** |
| 1b | **Reviewer** |
| 1c | **Audit Date** |
|  |  |
| **Reference (not for data entry)** | |
| 2a | **Gender** |
| 2b | **Age as of 8/4/2015** |
| 2c | **Date of Positive FIT (Index FIT)** |
| 2d | **Date of Colonoscopy Referral** |
| 2e | **Date of Colonoscopy Result** |
| 2f | **Date of Colonoscopy Health Maintenance Update** |
| 2g | **Date of Colonoscopy Health Maintenance Through** |
|  |  |
| **FIT/FOBT Order and Result** | |
|  | *Index FIT: first positive after 2/4/14* |
| 3a | **Had a FIT/FOBT after 2/4/2014?** |
| 3b | **Order code** |
|  | *See Order_Codes tab for expected values* |
| 3c | **Order Date** |
| 3d | **Ordering Clinic** |
| 3e | **FIT Kit Name (ie, OCMicro, Insure) or Description (ie, 2-kit)** |
|  |  |
| 3f | **Resulted (Y/N)** |
| 3g | **Resulted Date** |
| 3h | **Resulted value** |
|  |  |
| **Colonoscopy Referral** | |
|  | *Priority:  (1) First referral within 6 months prior to Index FIT through 1 year after Index FIT (2) If no referral in time period (1), then most recent referral* |
| 5a | **Had a Colonoscopy referral after 2/4/2014?** |
|  |  |
| 5b | **If no, reason for no referral:** |
|  |  |
|  | *If yes:* |
| 5c | **Referral Date** |
| 5d | **Referring Clinic** |
| 5e | **Referral Code** |
|  | *See Order_Codes tab for expected values* |
|  |  |
| **Colonoscopy Procedure** | |
|  | *Priority:  (1) First colonoscopy in 1 year after Index FIT (2) If no colonoscopy in time period (1), then most recent colonoscopy* |
| 6a | **Had a Colonoscopy after 2/4/2014?** |
|  |  |
| 6b | **If no, reason for no procedure:** |
|  |  |
|  | *If yes:* |
| 6c | **Colonoscopy Date** |
| 6d | **Colonoscopy Facility** |
|  |  |
|  | **Where documented (select all that apply)** |
| 6e | **Health Maintenance** |
| 6f | **Problem list** |
| 6g | **Surgical history** |
| 6h | **Scanned procedure report** |
| 6i | **Scanned pathology report** |
| 6h | **Care Everywhere procedure report** |
| 6i | **Care Everywhere pathology report** |
| 6j | **Other (describe)** |
|  |  |
| 6k | **Number of biopsies (enter 0 if none)** |
|  |  |
| **Pathology Report (from colonoscopy above)** | |
| 7a | **Colonoscopy pathology report in the chart?** |
|  |  |
|  | *If yes:* |
| 7b | **Pathology Date** |
|  |  |
| 7c | **Cancer** |
|  |  |
| 7d | **Cancer stage (most advanced)** |
|  |  |
| 7e | **Advanced adenoma** |
|  | *Any of the following:* |
|  | *A villous or tubulovillous adenoma of any size* |
|  | *An adenoma with "high grade dysplasia" of any size* |
|  | *A serrated adenoma >10 mm in size* |
|  | *A tubular adenoma >10 mm in size* |
|  | *3 or more serrated or tubular adenomas <10 mm in size.* |
|  |  |
| 7f | **Non-advanced adenoma** |
|  | *Any of the following:* |
|  | *Tubular adenoma < 10 mm in size* |
|  | *Serrated adenoma < 10 mm in size* |
|  |  |
| 7g | **Other polyps** |
|  |  |
| 7h | **No polyps/adenomas/cancers found on any biopsy** |
|  |  |
| **Abstractor Notes** | |
| 8a | **Additional Notes** |
|  | *optional* |
|  |  |

# Supplemental Table 2. Diagnosis codes (ICD-9 or -10) used

**Anticoagulant use**

ICD-9 V58.61 Long-term (current) use of anticoagulants

ICD-10 Z79.0 Long term (current) use of anticoagulants and antithrombotics/antiplatelets

**Proxy for NSAID use, derived from Evidex ^36^**

ICD-9 714 Rheumatoid arthritis

ICD-9 715 Osteoarthrosis and allied disorders

ICD-9 720 Ankylosing spondylitis

ICD-9 625.3 Dysmenorrhea

ICD-9 346 Migraine

ICD-10 M05, M06 Rheumatoid arthritis

ICD-10 M15, M16, M17, M18, M19 Osteoarthritis

ICD-10 M45 Ankylosing spondylitis

ICD-10 N94.4, N94.5, N94.6 Primary dysmenorrhea

ICD-10 G43 Migraine

**Hemorrhoids, diverticula, or anal fissure recorded prior to positive FIT**

ICD-9 455 Hemorrhoids

ICD-9 562 Diverticula of intestine

ICD-9 565.0 Anal fissure

ICD-10 K64 Hemorrhoids and perianal venous thrombosis

ICD-10 K57 Diverticular disease of intestine

ICD-10 K60.0, K60.1, K60.2 Anal fissure

**Diabetes and hypertension ICD 9 Codes, using “Elixhauser’s Original ICD-9-CM” ^35^**

ICD9 401.1, 401.9, 402.10, 402.90, 404.10, 404.90, 405.1, 405.9 Hypertension

ICD10 I10.x, I11.x-13.x, I15.x Hypertension

ICD9 250.0-250.3, 250.4-250.7, 250.9 Diabetes

ICD10 E10.0, E10.1, E10.2-E10.8, E10.9, E11.0, E11.1, E11.2-E11.8, E11.9, E12.0, E12.1, E12.2-E12.8, E12.9, E13.0, E13.1, E13.2-E13.8, E13.9, E14.0, E14.1, E14.2-E14.8, E14.9 Diabetes

# Supplemental Table 3. Factors associated with a false-positive FIT (i.e., no evidence of advanced neoplasia or any adenoma), N=1,027

|  | Unadjusted† | | | |  | Adjusted† | | |
| --- | --- | --- | --- | --- | --- | --- | --- | --- |
|  | OR | 95% CI | P |  | | OR | 95% CI | P |
| FIT type |  |  | 0.22 |  | |  |  | 0.36 |
| Hemosure vs Insure | 1.98 | (0.81-4.85) |  |  | | 1.87 | (0.65-5.38) |  |
| OC-Micro vs Insure | 1.28 | (0.73-2.23) |  |  | | 1.28 | (0.65-2.53) |  |
| Hemosure vs OC-Micro | 1.55 | (0.62-3.88) |  |  | | 1.46 | (0.50-4.26) |  |
| Age |  |  | 0.40 |  | |  |  |  |
| 50-64 | Ref |  |  |  | |  |  |  |
| 65-74 | 0.87 | (0.63-1.21) |  |  | |  |  |  |
| Female | 1.56 | (1.21-2.00) | 0.0005 |  | | 1.48 | (1.15-1.91) | 0.003 |
| Hispanic | 1.36 | (0.90-2.05) | 0.15 |  | |  |  |  |
| Non-white | 1.29 | (0.92-1.82) | 0.14 |  | |  |  |  |
| Language |  |  | 0.15 |  | |  |  | 0.33 |
| English | Ref |  |  |  | | Ref |  |  |
| Spanish | 1.60 | (0.99-2.59) |  |  | | 1.45 | (0.88-2.37) |  |
| Other | 1.02 | (0.69-1.51) |  |  | | 0.99 | (0.67-1.48) |  |
| Insurance status |  |  | 0.20 |  | |  |  | 0.35 |
| Medicaid | Ref |  |  |  | | Ref |  |  |
| Medicare | 0.81 | (0.57-1.15) |  |  | | 0.79 | (0.56-1.13) |  |
| Uninsured | 1.21 | (0.90-1.64) |  |  | | 1.12 | (0.82-1.53) |  |
| Commercial | 0.93 | (0.61-1.42) |  |  | | 0.89 | (0.58-1.36) |  |
| Federal Poverty Level |  |  | 0.46 |  | |  |  |  |
| <100% | Ref |  |  |  | |  |  |  |
| 100-150% | 0.97 | (0.68-1.40) |  |  | |  |  |  |
| >150% | 1.23 | (0.87-1.74) |  |  | |  |  |  |
| Unknown | 0.90 | (0.65-1.24) |  |  | |  |  |  |
| Co-morbidities |  |  |  |  | |  |  |  |
| Diabetes | 0.91 | (0.69-1.20) | 0.49 |  | |  |  |  |
| Hypertension | 0.88 | (0.69-1.14) | 0.34 |  | |  |  |  |
| Colorectal condition**‡** | 1.54 | (0.95-2.50) | 0.08 |  | | 1.52 | (0.93-2.49) | 0.09 |
| Anticoagulant use | 0.75 | (0.36-1.56) | 0.44 |  | |  |  |  |
| NSAIDs use | 1.22 | (0.91-1.65) | 0.19 |  | | 1.11 | (0.82-1.51) | 0.51 |
| Tobacco use |  |  | 0.31 |  | |  |  |  |
| Never | Ref |  |  |  | |  |  |  |
| Former | 0.86 | (0.63-1.18) |  |  | |  |  |  |
| Current | 0.77 | (0.56-1.05) |  |  | |  |  |  |
| Unknown | 1.06 | (0.68-1.63) |  |  | |  |  |  |
| Season of FIT return |  |  | 0.07 |  | |  |  |  |
| Winter | Ref |  |  |  | |  |  |  |
| Spring | 0.72 | (0.66-1.35) |  |  | |  |  |  |
| Summer | 1.12 | (0.53-0.99) |  |  | |  |  |  |
| Fall | 0.94 | (0.66-1.35) |  |  | |  |  |  |

† All models include a random effect for health center, nested within FIT brand, to account for any unmeasured population differences across center. The adjusted model additionally includes FIT kit type and all variables with unadjusted p<0.20.

**‡** From diagnosis codes present in the EHR within two years prior to FIT eligibility (Supplemental Table 2).

# Supplemental Figure 1. PPVs by FIT type for the main outcome of advanced neoplasia (left) and for the sensitivity analysis that included any adenoma (right)
